# Augmented-Medication CardioPulmonary Resuscitation Trials in out-of-hospital cardiac arrest: a pilot randomized controlled trial

**DOI:** 10.1186/s13054-022-04248-x

**Published:** 2022-12-07

**Authors:** June-sung Kim, Seung Mok Ryoo, Youn-Jung Kim, Chang Hwan Sohn, Shin Ahn, Dong Woo Seo, Seok In Hong, Sang-Min Kim, Bora Chae, Won Young Kim

**Affiliations:** Department of Emergency Medicine, University of Ulsan College of Medicine, Asan Medical Center, 88 Olympic-Ro-43-Gil, Songpa-gu, Seoul, 138-736 Republic of Korea

**Keywords:** Resuscitation, Out-of-hospital cardiac arrest, Vasopressin, Epinephrine

## Abstract

**Background:**

Previously conducted physician-centered trials on the usefulness of vasopressin have yielded negative results; thus, patient-oriented trials have been warranted. We hypothesize that Augmented-Medication CardioPulmonary Resuscitation could be helpful for selected patients with out-of-hospital cardiac arrest (OHCA).

**Methods:**

This is a double-blind, single-center, randomized, placebo-controlled trial conducted in the emergency department in a tertiary, university-affiliated hospital in Seoul, Korea. A total of 148 adults with non-traumatic OHCA who had initial diastolic blood pressure (DBP) < 20 mm Hg via invasive arterial monitoring during the early cardiac compression period were randomly assigned to two groups. Patients received a dose of 40 IU of vasopressin or placebo with initial epinephrine. The primary endpoint was a sustained return of spontaneous circulation. Secondary endpoints were survival discharge, and neurologic outcomes at discharge.

**Results:**

Of the 180 included patients, 32 were excluded, and 148 were enrolled in the trial. A sustained return of spontaneous circulation was achieved by 27 patients (36.5%) in the vasopressin group and 24 patients (32.4%) in the control group (risk difference, 4.1%; *P* = .60). Survival discharge and good neurologic outcomes did not differ between groups. The trial group had significantly higher median DBPs during resuscitation than the control group (16.0 vs. 14.5 mm Hg, *P* < 0.01). There was no difference in end-tidal carbon dioxide, acidosis, and lactate levels at baseline, 10 min, and end-time.

**Conclusion:**

Among patients with refractory vasodilatory shock in OHCA, administration of vasopressin, compared with placebo, did not significantly increase the likelihood of return of spontaneous circulation.

**Supplementary Information:**

The online version contains supplementary material available at 10.1186/s13054-022-04248-x.

## Introduction

Among resuscitative steps of out-of-hospital cardiac arrest (OHCA), prompt vasopressor administration is emphasized to increase vital organ perfusion pressure during cardiopulmonary resuscitation (CPR) [1–3]. Although epinephrine plays a key role, it’s effectiveness would be limited because of catecholamine-related side effects [4, 5]. Vasopressin is another candidate, which stimulates smooth muscle vasoconstriction without the catecholamine effect [6]. However, trials have shown no benefit from vasopressin for return of spontaneous circulation (ROSC), or neurologic outcomes, over the standard dose of epinephrine [7–10]. Previous reports about the usefulness of vasopressin during resuscitation have primarily been physician-oriented; therefore, a patient-centered analysis is warranted.

Coronary perfusion pressure is the gap between aortic and right atrial relaxation pressure during cardiopulmonary resuscitation, which correlates with myocardial blood flow and the quality of resuscitation [11]. We designed the Augmented-Medication CardioPulmonary Resuscitation (AMCPR) trial to evaluate the potential for vasopressin to increase diastolic blood pressure (DBP) and improve the achievement of ROSC for adult patients with non-traumatic OHCA. We hypothesized that additive vasopressin to epinephrine would be effective for patients with OHCA and those with low DBP.

## Methods

### Trial design

We performed an investigator-initiated, single-center, prospective, placebo-controlled, double-blind, superiority, randomized clinical trial of additive vasopressin during resuscitation of adult OHCA patients between August 2017 and August 2021. Emergency Medical Services in South Korea usually do not terminate resuscitation at the scene. They provided advanced CPR according to guidelines, including administration of epinephrine, and placement of extra-glottic device, and transported nearby hospital as soon as possible. After resuscitation, the physicians on duty provided detailed information about the study to patient caregivers. The Institutional Review Board waived the requirement for written consent because of the urgent administration of trial drugs (number: 2017-0669). The study was registered at clinicaltrials.gov (NCT03191240) before enrollment.

### Participants

Patients were included if they were: non-traumatic, non-sustained, shockable adult (≤ 19 years old) patients with OHCA who had successfully accessed invasive arterial catheter within 6 min (3 cycles) after presenting at the emergency department with DBP less than 20 mm Hg. The exclusion criteria are reported in Additional file 1: Table S1.

### Randomization and intervention

Additional file 1: Fig. S1 presents the brief study protocol [12]. Emergency medicine physicians on duty tried to access the radial or femoral artery percutaneously using Arterial Leadercath (Vygon, France) within 6 min of admission of all eligible patients. Correct placement of the catheter in the artery was confirmed by emergency medicine physicians using bedside ultrasonography and square waveform test. When initial DBPs were below 20 mm Hg, the trial-participating nurse opened a pre-made, concealed, uniquely numbered, but otherwise identical-appearing card contained a word (i.e., vasopressin or saline). Patients were randomly assigned in a 1:1 ratio to inject vasopressin or placebo with epinephrine using random permuted blocks of sizes 2 and 4, stratified by study site at enrollment. The nurse administrated vasopressin 40 IU or normal saline immediately after the epinephrine injection. The remaining medical personnel who participated in the resuscitation were blinded to the infusion of vasopressin or saline. If ROSC was not achieved within 3 min, the same dose of vasopressin or saline was administered after an epinephrine bolus (i.e., total dose of vasopressin was up to 80 IU).

### Outcomes

The primary outcome was sustained ROSC, defined as a palpable carotid pulse lasting more than 20 min. The secondary outcomes were survival discharge and good neurologic recovery.

### Statistical analysis

This study was conducted to assess efficacy using a composite of end points, in which the rate of sustained ROSC in the control group was 21% according to previous researches [21]. Sample size calculation was conducted with an expected difference of 25%. For *a* = 0.05 and statistical power = 0.80, a total sample size of 74 patients was required in each group. The categorical variables were presented as a number and percentage, and continuous variables were displayed as a median and interquartile range (IQR) because of the non-normal distributions. The primary and secondary outcomes of the study were analyzed by the Chi-square test, Fisher’s exact test, Student’s *t*-test, or the Mann–Whitney U test, as appropriate. Statistical significance was considered at a *P*-value of < 0.05. All statistical analyses were conducted using SPSS (IBM SPSS, Version 27.0; IBM Corporation, Armonk, New York).

## Results

**Fig. 1:**
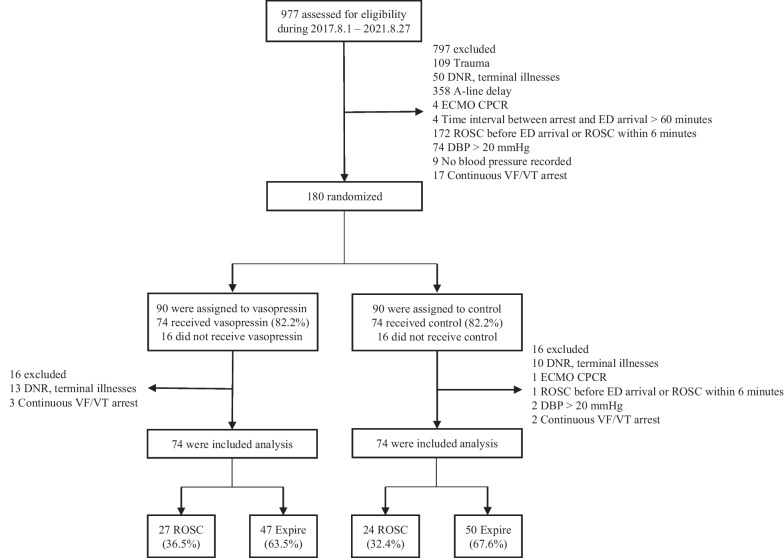
Study flowchart. *DNR* do-not-resuscitate, *ECMO* extracorporeal membrane oxygenation, *CPCR* cardiopulmonary cerebral resuscitation, *ED* emergency department, *ROSC* return of spontaneous circulation, *DBP* diastolic blood pressure, *VF* ventricular fibrillation, *VT* ventricular tachycardia

After randomization of 180 patients, 16 (17.8%) for each group were excluded from the analysis (Fig. 1). Data from the remaining 148 patients were analyzed; 74 received vasopressin combined with epinephrine, and 74 received epinephrine only.

Baseline characteristics are presented in Table 1. The median age was 77 years, with a greater proportion of males, and the two groups showed a similar proportion of comorbidities. Non-trial-related interventions after ROSC, including percutaneous coronary intervention, target temperature management, and renal replacement therapy, were also similar between groups.

**Table 1:** Baseline characteristics according to treatment assignment

Characteristics	Total (*n* = 148)	AMCPR (*n* = 74)	Placebo (*n* = 74)
*Patients characteristics*			
Age, year	77.0 (68.3–83.0)	77.5 (70.0–83.0)	77.0 (67.0–83.0)
Sex			
Male	100 (67.6)	47 (63.5)	53 (71.6)
Female	48 (32.4)	27 (36.5)	21 (28.4)
*Comorbidities*			
Coronary artery syndrome	22 (14.9)	10 (13.5)	12 (16.2)
Atrial fibrillation	7 (4.7)	3 (4.1)	4 (5.4)
Chronic heart failure	5 (3.4)	2 (2.7)	3 (4.1)
Stroke	9 (6.1)	4 (5.4)	5 (6.8)
Hypertension	64 (43.2)	31 (41.9)	33 (44.6)
Diabetes	49 (33.1)	25 (33.8)	24 (32.4)
Pulmonary disease	14 (9.5)	3 (4.1)	11 (14.9)
Neurologic disease	18 (12.2)	10 (13.5)	8 (10.8)
Kidney disease	11 (7.4)	6 (8.1)	5 (6.8)
Liver disease	2 (1.4)	1 (1.4)	1 (1.4)
Cancer	21 (14.2)	9 (12.2)	12 (16.2)
*Cardiac arrest characteristics*			
Witnessed	92 (62.2)	47 (63.5)	45 (60.8)
Bystander chest compression	91 (61.5)	46 (62.2)	45 (60.8)
Prehospital AED	13 (8.8)	9 (12.2)	4 (5.4)
*Initial rhythm*			
Asystole	106 (71.6)	50 (67.6)	56 (75.7)
Pulseless electrical activity	32 (21.6)	17 (23.0)	15 (20.3)
Ventricular fibrillation ^a^	9 (6.1)	6 (8.1)	3 (4.1)
*Presumed arrest cause*			
Cardiac	77 (52.7)	37 (50.7)	40 (54.8)
Other medical	64 (43.8)	34 (46.6)	30 (41.1)
Prehospital low flow time, min	28.0 (23.0–34.0)	29.0 (25.0–33.3)	26.0 (21.0–34.0)
*Time from ED arrival to*			
Epinephrine administration, min	1.0 (1.0–2.0)	1.0 (1.0–3.0)	1.0 (0.0–2.0)
Vasopressin or normal saline administration, min	4.0 (3.0–6.0)	5.0 (3.0–5.0)	4.0 (3.0–6.0)
*Treatment after ROSC*			
PCI	9 (6.1)	5 (6.8)	4 (5.4)
TTM	34 (23.0)	16 (21.6)	18 (24.3)
RRT	9 (6.1)	4 (5.4)	5 (6.8)

Data presented as number (percentage) and median (interquartile range)

*AMCPR* Augmented-Medication of CardioPulmonary Resuscitation; *AED* automated external defibrillator; *ED* emergency department; *ROSC* return of spontaneous circulation; *PCI* percutaneous coronary intervention; *TTM* target temperature management; *RRT* renal replacement therapy

^a^Patients with ventricular fibrillation at initial presentation which converted to pulseless electrical activity or asystole within 6 min (3 cycles) were included

The achievement rates of sustained ROSC were not different between AMCPR and the placebo group (36.5 vs. 32.4%; absolute risk difference 4.1%; relative risk [95% CI] 0.94 [0.74–1.19]); *P* = 0.60) (Table 2). Moreover, survival discharge, good neurologic recovery at discharge, and acidosis were not significantly different between the groups. Additional file 1: Table S2 presents the differences between the two groups in DBP, ETCO_2_, acidosis, and lactate levels.

**Table 2:** Outcomes according to treatment assignment

	Total (*n* = 148)	AMCPR (*n* = 74)	Placebo (*n* = 74)	Difference (%)	Relative risk (95% CI)	*P*
*Primary outcome*						
Sustained ROSC	51 (34.5)	27 (36.5)	24 (32.4)	4.1	0.94 (0.74–1.19)	0.60
*Secondary outcomes*						
Survival discharge	12 (8.1)	6 (8.1)	6 (8.1)	0	1.00 (0.91–1.10)	1.00
Good neurologic recovery^a^	0 (0.0)	0 (0.0)	0 (0.0)	0	1.00 (1.00–1.00)	1.00

Data presented as the median (interquartile range)

*AMCPR* Augmented-Medication of CardioPulmonary Resuscitation; *ROSC* return of spontaneous circulation; *CI* confidence interval

^a^Cerebral Performance Category 1 or 2 was considered a favorable outcome

**Fig. 2:**
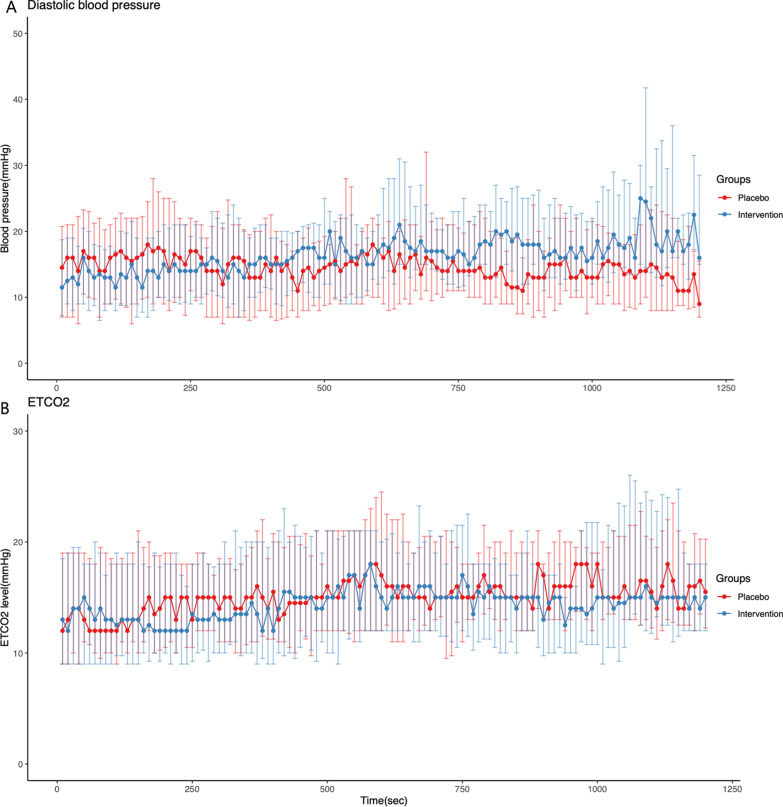
Trends of diastolic blood pressure **A** and end-tidal carbon dioxide **B** levels during resuscitation according to intervention

Figure 2 presents the trends in DBPs and ETCO_2_ levels during resuscitation. The DBP of the AMCPR group tent to be higher compared with the placebo group, which was evident after 12.5 min after the administration of vasopressin. Meanwhile, augmented medication of vasopressin could not improve ETCO_2_ levels.

## Discussion

In this randomized trial, we found that augmented medication of vasopressin did not enhance the rate of sustained ROSC, survival discharge, and recovery of favorable neurologic outcome among selected patients with DBP lower than 20 mm Hg, despite elevating DBP within 10 min of drug administration. This implied that the AMCPR protocol did not improve the outcomes of OHCA patients.

Despite reports from a recent trial that patients with in-hospital cardiac arrest showed a significantly increased rate of ROSC in the intervention (vasopressin plus methylprednisolone) group, our study did not find any improvement, which could be attributed to several possibilities [13]. First, the severity of the included patients was poorer than the average population with OHCA. Previously well-known favorable factors, such as shockable rhythm, prehospital ROSC, and initial DBP above 20 mm Hg, were excluded from our trial because of the study design [14]. Second, the timing of the injection could have been too delayed to improve outcomes. Owing to the outpatient setting, vasopressin was administered after ED arrival, not at the scene. The previous trial also reported that the usefulness of vasopressin decreased in prolonged resuscitation, and late timing of drug infusion could have diminished the true effect of vasopressin in our study [15]. Lastly, the total amount of vasopressin might not have been enough to increase coronary perfusion pressure. Animal and observational studies revealed a positive correlation between the serum level of vasopressin and the rate of ROSC [16]. We could not measure endogenous vasopressin levels in patients with OHCA, and it could be possible that 40 or 80 IU of vasopressin was insufficient to achieve ROSC.

The strong point of our trial was that patient-centered inclusions and interventions among patients with OHCA were conducted by real-time invasive monitoring of DBP. Although the guidelines for CPR have recommended monitoring to tailor CPR quality, the precise protocol and numeric targets have not yet been decided due to a paucity of evidence. Arterial catheter placement intra-cardiac arrest is ubiquitous in the intensive care unit setting; however, it is not yet widespread in the emergency department [17]. Recent studies with children in intensive care units who already had invasive arterial BP monitoring and in-hospital cardiac arrest reported that mean DBP above 25–30 mm Hg was associated with survival discharge and good neurologic outcomes [18].

There are several limitations to our study. First, this trial was performed in single, urban hospital and could not be generalized in other circumstances. Second, the most common cause of exclusion was arterial line insertion delayed after 6 min during resuscitation (*n* = 358, 36.6%); excluding patients in whom arterial catheter placement was difficult could lead to selection bias. Third, hidden confounders, especially quality of prehospital resuscitation performed by Emergency Medical Services, could effect on the results even after randomization. Fourth, relatively small sample size could decrease statistical power. When we designed the study, we calculated sample size with absolute increase (from 21 to 46%) of trial drug because vasopressin would be a great role in epinephrine-refractory patients. However, relative increasing (from 21 to 25%) was more appropriate which need about 1500 patients for proving our hypothesis.

## Conclusion

In patients with low DBP in initial resuscitation, additive vasopressin did not help enhance the ROSC rate, survival discharge, or good neurologic recovery; however, vasopressin could increase coronary perfusion pressure during CPR.

## Supplementary Information

**Additional file 1**. **Table S1.** Exclusion criteria. **Figure S1.** Study protocol. **Table S2**. DBP, ETCO_2_, acidosis, and lactic clearance during resuscitation.

## Data Availability

The datasets are available from corresponding author on reasonable request.
