# Supplementary material for: Augmented-Medication CardioPulmonary Resuscitation Trials in out-of-hospital cardiac arrest: a pilot randomized controlled trial
Source: Crit Care. 2022 Dec 7;26:378. doi: 10.1186/s13054-022-04248-x (PMC9727995; doi:10.1186/s13054-022-04248-x)
Supplement: Supplementary file 1 — Additional file 1. Table S1. Exclusion criteria. Figure S1. Study protocol. Table S2. DBP, ETCO2, acidosis, and lactic clearance during resuscitation. [file 13054_2022_4248_MOESM1_ESM.pdf]

Table S1. Exclusion criteria

(1) premature termination of resuscitation due to do-not-resuscitation order or terminal-stage illness without an active treatment plan<br>(2) arterial catheter insertion delay or fail (≥ 6 minutes)<br>(3) successful return of spontaneous circulation before hospital arrival or ROSC within 6 minutes<br>(4) Diastolic blood pressure above 20 mmHg during entire resuscitation<br>(5) extracorporeal membrane oxygenation cardiac pulmonary resuscitation<br>(6) delayed emergency department presentation (≥ 60 minutes)<br>(7) Continuous ventricular fibrillation or ventricular tachycardia (≥ 6 minutes from initial presentation or sustained remained resuscitation after enrollment)

Figure S1. Study protocol

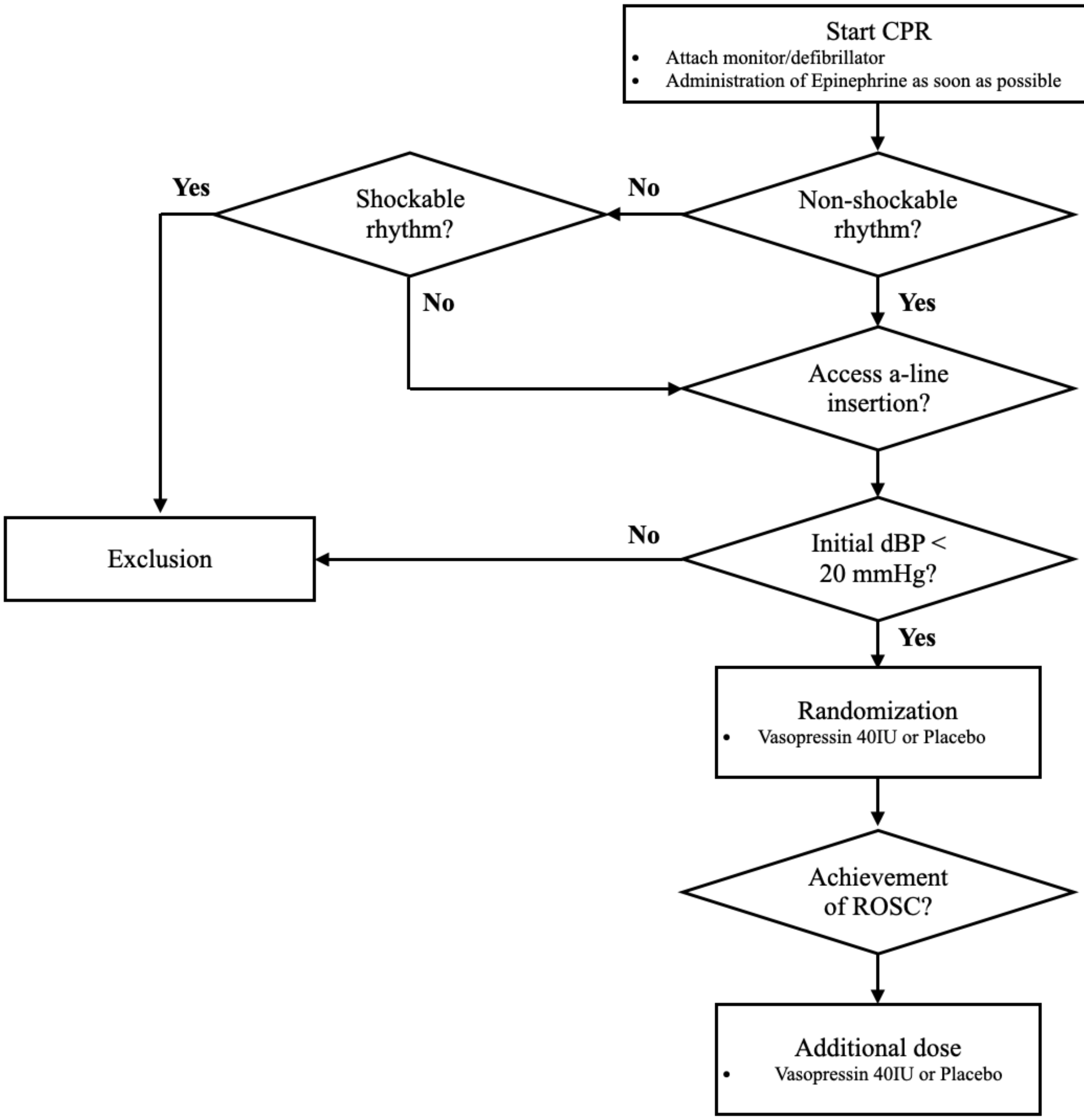

After inserting a-line catheter, dBP and end-tidal carbon dioxide were recorded by using smartphone (Galaxy S, Samsung, Korea) and extracted each data every 10 seconds. Arterial blood gas analyses were conducted just after a-line insertion and repeated every 10 minutes.

Abbreviations: CPR, cardiopulmonary resuscitation; a-line, arterial line; ROSC, return of spontaneous circulation.

Table S2. DBP, $ETCO_2$, acidosis, and lactic clearance during resuscitation

Characteristics	Total (n = 148)	AMCPR (n = 74)	Placebo (n = 74)	*P*
DBP, mmHg	15.25 (14.00 – 17.00)	16.00 (15.00 – 18.00)	14.50 (13.50 – 16.00)	< 0.01
$ETCO_2$, mmHg	15.00 (14.00 – 15.50)	14.75 (13.50 – 15.00)	15.00 (14.00 – 16.00)	< 0.01
pH at initial	6.84 (6.72 – 6.96)	6.86 (6.73 – 7.01)	6.83 (6.70 – 6.95)	0.24
pH at end	6.88 (6.74 – 7.02)	6.92 (6.73 – 7.03)	6.86 (6.75 – 7.03)	0.92
Lactate at initial, mmol/L	12.80 (9.65 – 15.00)	11.25 (8.83 – 14.43)	13.55 (10.60 – 15.00)	0.02
Lactate at end, mmol/L	13.50 (10.25 – 15.00)	12.65 (10.10 – 14.85)	13.60 (10.25 – 15.00)	0.41

Data presented as the median (interquartile range).

Abbreviations: AMCPR, Augmented-Medication of CardioPulmonary Resuscitation; DBP, diastolic blood pressure; $ETCO_2$, end-tidal carbon dioxide.
